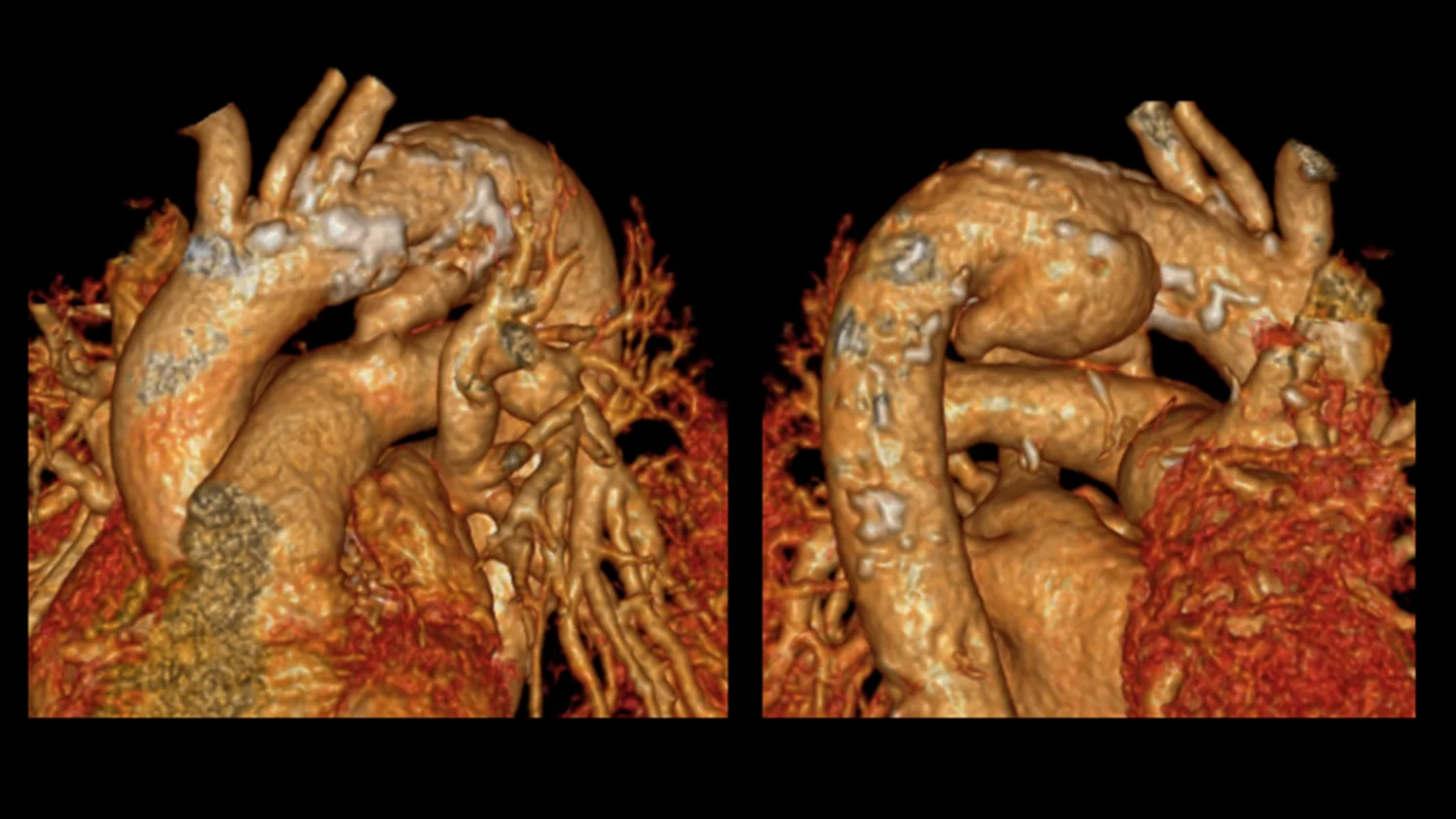# Aortopulmonary fistula complicating a ruptured, infected proximal descending thoracic aortic pseudoaneurysm

**DOI:** 10.1016/j.jvscit.2026.102552

**Published:** 2026-10-09

**Authors:** Salomé Kuntz, Jonathan Grandhomme, Thomas Lemmet, Nicolas Lefebvre, Anne Lejay, Nabil Chakfé

**Affiliations:** a Department of Vascular Surgery Kidney Transplantation and Innovation, Strasbourg University Hospitals, Strasbourg, France; b Gepromed, Strasbourg, France; c Strasbourg Biomedicine Center Research (CRBS), Strasbourg, France; d Department of Infectious Disease, Strasbourg University Hospitals, Strasbourg, France

Aortopulmonary fistulas (APFs) are rare, potentially fatal communications between the aorta and the pulmonary vasculature.^1^ Infectious aortic pseudoaneurysms are an exceptional cause.^2^

A 76-year-old man with smoking history and dyslipidemia presented with syncope following several days of chest pain, dyspnea, cough with sputum production, and dysphagia. He had no fever, with leukocytosis (15 G/L) and elevated C-reactive protein (56 mg/L). Computed tomography angiography (CTA) demonstrated a ruptured zone 3 saccular thoracic aortic pseudoaneurysm communicating with the pulmonary artery. Three-dimensional CTA reconstructions delineated the pseudoaneurysm (*A*, Cover), whereas cross-sectional CTA demonstrated the APF (*B*, *left*).

Blood cultures obtained preoperatively grew methicillin-susceptible *Staphylococcus aureus*. No definite portal of entry was identified, although a respiratory source was suspected. Transthoracic echocardiography showed no evidence of endocarditis.

Emergency endovascular repair was performed in zone 3, with deployment of a 31 × 31 × 100 mm thoracic stent-graft (GORE TAG Thoracic Endoprosthesis; W. L. Gore & Associates), without left subclavian artery coverage achieving exclusion of the pseudoaneurysm.^3^, ^4^ Given the low pressure of the pulmonary circulation, the residual pulmonary pseudoaneurysm was managed conservatively (*B*, *right*).

Empirical amoxicillin-clavulanate therapy was subsequently replaced with targeted intravenous cloxacillin and rifampicin for 6 weeks, followed by oral trimethoprim-sulfamethoxazole and rifampicin, for a total antimicrobial treatment duration of 3 months.

Given the patient’s age, frailty, high-operative risk, and favorable clinical and biological response, secondary open graft explantation was not pursued after multidisciplinary discussion.^5^ Antibiotics were discontinued following multidisciplinary assessment and normalization of inflammatory markers and normal CTA.

The residual pulmonary pseudoaneurysm decreased in size from 51 mm postoperatively (*B*, *right*) to 40 mm at 3 years (*C*). Annual CTA showed no recurrence. Infectious disease follow-up continued for 2 years, and positron emission tomography at 2 years showed no evidence of infection.

Although the combination of methicillin-susceptible *S. aureus* bacteremia, inflammatory response, and aortic morphology strongly supported infectious aortitis, secondary infection of a pre-existing aortic lesion cannot be definitively excluded.

The patient provided written informed consent for publication.

This case demonstrates durable clinical and imaging remission at 3-year after emergency thoracic endovascular aortic repair and a finite course of targeted antimicrobial therapy in a high-risk patient with APF.

**Figure:**
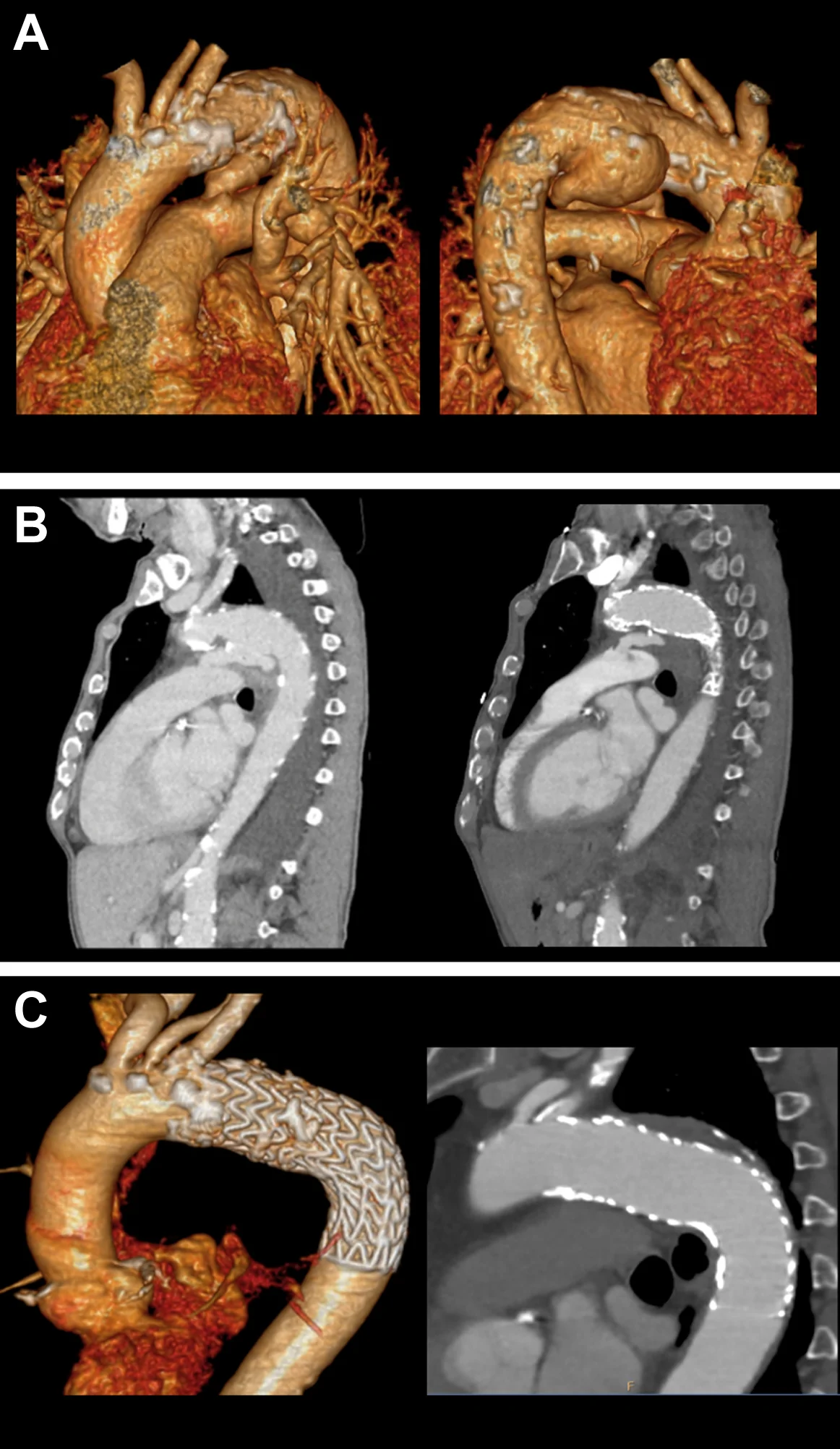

## Funding

None.

## Disclosures

None.

## Figures and Tables

**Cover image:**